# Supplementary material for: Co-Existing Vestibular Hypofunction Impairs Postural Control, but Not Frailty and Well-Being, in Older Adults with Benign Paroxysmal Positional Vertigo
Source: J Clin Med. 2025 Apr 14;14(8):2666. doi: 10.3390/jcm14082666 (PMC12027737; doi:10.3390/jcm14082666)
Supplement: Supplementary file 1 [file jcm-14-02666-s001.zip › jcm-3531384-supplementary.pdf]

Supplementary tables and figures

Supplementary figure S1. Flow chart of methods and selection process of participants

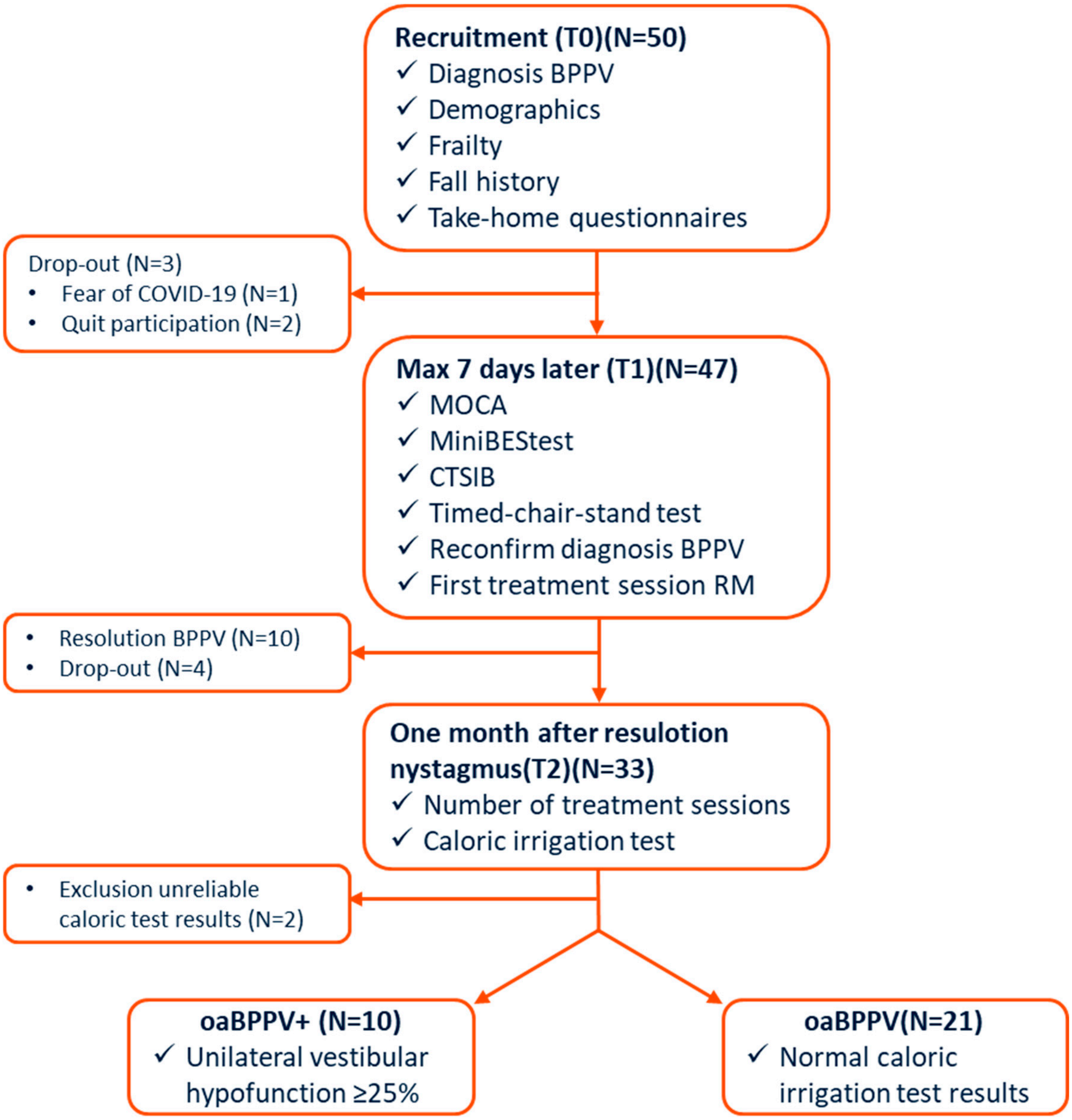

Abbreviations: BPPV, benign paroxysmal positioning vertigo; MOCA, montreal cognitive assessment; MiniBEStest, mini balance evaluation system test; CTSIB, Clinical Test of Sensory Interaction on Balance; RM; repositioning maneuver; oaBPPV+, older adults with BPPV and vestibular hypofunction; oaBPPV, older adults with BPPV

Supplementary table S1. Checklist of comorbidities

- ☐ Cardiovascular diseases *(Have you ever had a disorder of your heart or blood vessels?)*
- ☐ Cerebrovascular diseases *(Have you ever had a disorder in your brain?)*
- ☐ Diabetes Mellitus (type 1 or 2) *(Have you ever been diagnosed with diabetes?)*
- ☐ Hypertension *(Have you ever been diagnosed with hypertension?)*
- ☐ Hypercholesterolemia *(Do you have elevated cholesterol?)*
- ☐ Vitamin D deficiency (*Have you ever been diagnosed with vitamin d-deficiency*?)
- ☐ Osteoporosis *(Have you ever been diagnosed with osteoporosis?)*
- ☐ Others *(Do you have any other medical disorders?)*

Supplementary table S2. Number of repositioning manoeuvres needed for resolution of nystagmus according to BPPV diagnose.

BPPV diagnose	oaBPPV+	oaBPPV
PSCC	2(2.5)	3(2)
LSCC geotropic	5(9)	0
LSCC apogeotropic	0	1(/)

Medians(interquartile range) for number of repositioning maneuvers needed. Abbreviations: BPPV, benign paroxysmal positioning vertigo; PSCC, posterior semicircular canal BPPV; LSCC, lateral semicircular canal BPPV; oaBPPV+, older adults with BPPV and vestibular hypofunction; oaBPPV: older adults with BPPV.

Supplementary Figure S2. Results of the sub scores of the Dizziness Handicap Inventory

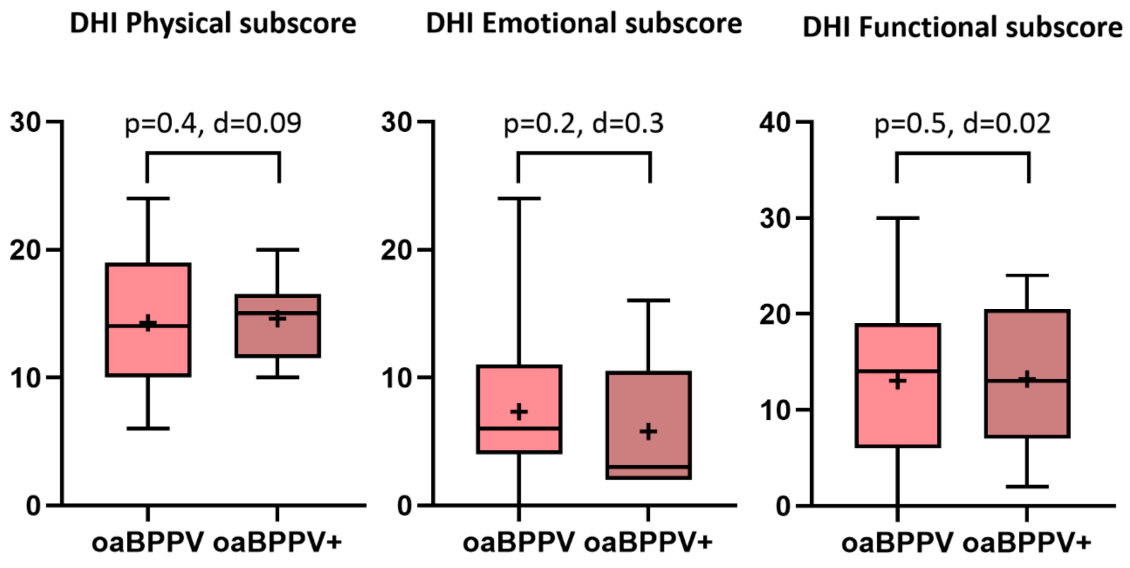

Supplementary Figure S2. Physical, emotional and functional subscores of the Dizziness Handicap Inventory in older adults with BPPV (n=21) and co-existing vestibular hypofunction canal paresis compared to older adults with BPPV (n=10). The boxplots display the medians, interquartile range, and minimum and maximum values, with the '+' symbol representing the mean values. Significant p-values after Holm-Bonferroni correction are indicated with '*'. A-breviations: oaBPPV+, older adults with BPPV and vestibular hypofunction; oaBPPV, older adults with BPPV; DHI, dizziness handicap inventory;

Supplementary table S3. Results of Clinical Test of Sensory Interaction on Balance

CTSIB	oaBPPV+	oaBPPV
CTSIB1		
Area (m^2/s^4)	0.02(0.03)	0.01(0.01)
Velocity (m/s)	0.1(0.2)	0.1(0.1)
Path (m/s^2)	5.2(1.8)	5.2(2.4)
Range (m/s^2)	0.3(0.3)	0.3(0.3)
Time (s)	30(0.01)	30(0)
CTSIB2		
Area (m^2/s^4)	0(0.02)	0.02(0.07)
Velocity (m/s)	0.2(0.2)	0.1(0.1)
Path (m/s^2)	8.7(6.5)	7.2(3.6)
Range (m/s^2)	0.5(0.4)	0.4(0.1)
Time (s)	30(0.01)	30(0.01)
CTSIB3		
Area (m^2/s^4)	0.4(0.1)	0.02(0.02)
Velocity (m/s)	0.1(0.2)	0.1(0.08)
Path (m/s^2)	7.9(11.4)	7.2(4.7)
Range (m/s^2)	0.5(0.7)	0.4(0.3)
Time (s)	30(0)	30(0.01)
CTSIB4		
Area (m^2/s^4)	0.1(0.5)	0.1(0.05)
Velocity (m/s)	0.2(0.09)	0.1(0.01)
Path (m/s^2)	7.5(20.3)	8.5(2.4)
Range (m/s^2)	0.6(0.1)	0.5(0.4)
Time (s)	30(0)	30(0)
CTSIB5		
Area (m^2/s^4)	0.7(2.8)	0.1(0.2)
Velocity (m/s)	0.2(0.3)	0.2(0.2)
Path (m/s^2)	40.6(153.2)	14.5(10.7)
Range (m/s^2)	1.8(2)	0.9(0.3)
Time (s)	18(25.3)	30(0.01)
CTSIB6		
Area (m^2/s^4)	0.7(1.9)	0.1(0.1)
Velocity (m/s)	0.3(0.3)	0.3(0.2)
Path (m/s^2)	26.8(52.3)	16.1(7.9)
Range (m/s^2)	1.6(3.3)	0.1(0.4)
Time (s)	24.120.9)	30(0.01)

Medians(interquartile range) for CTSIB. Abbreviations: CTSIB, Clinical Test of Sensory Interaction on Balance; CTSIB1, standing on a firm surface with eyes open; CTSIB2, standing on a firm surface with a visual dome; CTSIB3, standing on a firm surface with eyes closed; CTSIB4, standing on a foam surface with eyes open; CTSIB5, standing on foam surface with a visual dome; CTSIB6, standing on a foam surface with eyes closed; oaBPPV+, older adults with BPPV and vestibular hypofunction; oaBPPV: older adults with BPPV.
